# Anti-Inflammatory, Anti-Osteoclastogenic and Antioxidant Effects of *Malva sylvestris* Extract and Fractions: *In Vitro* and *In Vivo* Studies

**DOI:** 10.1371/journal.pone.0162728

**Published:** 2016-09-19

**Authors:** Bruna Benso, Marcelo Franchin, Adna Prado Massarioli, Jonas Augusto Rizzato Paschoal, Severino Matias Alencar, Gilson Cesar Nobre Franco, Pedro Luiz Rosalen

**Affiliations:** 1 Department of Physiological Sciences, Piracicaba Dental School, State University of Campinas, Piracicaba, SP, Brazil; 2 School of Dentistry, Faculty of Medicine, Universidad Austral de Chile, Valdivia, Chile; 3 Department of Agri-food Industry, Food and Nutrition, “Luiz de Queiroz” College of Agriculture, University of Sao Paulo, Piracicaba, SP, Brazil; 4 Department of Physics and Chemistry, School of Pharmaceutical Sciences of Ribeirao Preto, University of Sao Paulo, Riberao Preto, SP, Brazil; 5 Department of General Biology, Laboratory of Physiology and Pathophysiology, State University of Ponta Grossa, Ponta Grossa, PR, Brazil; University of British Columbia, CANADA

## Abstract

Given their medical importance, natural products represent a tremendous source of drug discovery. The aim of this study was to investigate *Malva sylvestris* L. extract and fractions and their pharmacological activities followed by chemical identification. The aqueous fraction (AF) was identified as the bioactive fraction in the *in vitro and in vivo* assays. The AF controlled the neutrophil migration to the peritoneal cavity by 66%, inhibited the antiedematogenic activity by 58.8%, and controlled IL-1β cytokine expression by 54%. The *in vitro* viability tests showed a concentration-dependent effect, where the MSE and fractions at concentrations under 10 μg/mL were non-toxic to cells. Transcriptional factors of carbonic anhydrase II (*CAII*), cathepsin K (*Ctsk*) and tartrate-resistant acid phosphatase (*TRAP*) were analyzed by qPCR in RAW 264.7 cell lines. The gene expression analysis showed that the AF was the only treatment that could downregulate all the study genes: *CAII*, *Ctsk* and *TRAP* (p<0.05). TRAP staining was used to evaluate osteoclast formation. AF treatments reduced the number of osteoclastogenesis 2.6-fold compared to the vehicle control group. Matrix metalloproteinase 9 (MMP-9) activity decreased 75% with the AF treatment. Moreover, the bioactive fraction had the ability to regulate the oxidation pathway in the ABTS (2,2-Azino-bis (3-ethylbenzthiazoline-6-sulfonic acid) assay with an activity equivalent to 1.30 μmol Trolox/g and DPPH (2,2-diphenyl-1-picrylhydrazyl) radicals 1.01 g/L. Positive ion ESI-mass spectrometry for molecular ions at m/z 611 and 633 confirmed rutin as the major compound in the AF. The AF of *M*. *sylvestris* presented anti-inflammatory, controlled osteoclastogenic mechanisms and antioxidant abilities in different *in vitro* and *in vivo* methods. In addition, we suggest that given its multi-target activity the bioactive fraction may be a good candidate in the therapy of chronic inflammatory diseases.

## Introduction

Inflammation, a biological process that involves vascular and cellular events coordinated by mediators like prostaglandin, leukotrienes, cytokines and thromboxanes that include an essential and protective mechanism of the organism in response to injury, infection and trauma [1,2]. However, prolonged inflammation may lead to chronic diseases including rheumatoid arthritis and periodontitis, which are associated with tissue injury and bone resorption [2]. During the pathogenesis, pro-inflammatory mediators and reactive oxygen species (ROS) can promote osteoblast apoptosis and bone resorption through the activation of NF-κB signaling [3]. The classic pathway for NF-κB stimulation includes an upregulation of RANKL, an osteoclastogenic cytokine that induces bone resorption by promoting osteoclast differentiation and activation [4].

Three peptides and super families RANKL, RANK and OPG regulate the activation and differentiation of osteoclasts [5]. RANKL plays an essential role in the differentiation, recruitment, activation, and survival of osteoclasts by binding to its receptor (RANK) on osteoclasts or progenitor cells [6]. Studies showed that sRANKL and macrophage colony-stimulating factors (CSF-1/MCSF) factors have the ability to stimulate osteoclast differentiation from peripheral blood cell precursors [7]. A number of the RANK-induced signaling pathways in osteoclasts ultimately induce the expression of enzymes involved in regulating the dissolution of mineral and collagen, including carbonic anhydrase (CAII), cathepsin K (Ctsk) and tartrate-resistant acid phosphatase (TRAP) [8,9]. The main role of RANKL is the signaling and the regulation of homeostasis leading to the system to health or disease state. [7].

In addition, matrix metalloproteinases (MMP) are a family of proteolytic enzymes involved in extracellular matrix degradation that comprise bone and matrix [6]. Of this group, MMP-9 is an important proteinase that osteoclasts express at high levels. Studies show a correlation between MMP-9 enzyme activity and the tissue/bone destruction levels in chronic immune-mediated inflammatory diseases [9,10].

Traditionally, anti-inflammatory therapy has focused on the modulation of pro-inflammatory mediators and/or adhesion molecule expression [11,12]. However, in the past few years it has been recognized that the inflammation resolution may be based on multi-target drugs [13]. Multiple signaling pathways are a way to improve the pro-inflammatory, immunomodulatory and pro-resolving cascades, which define the aspects of the inflammation [14].

In this context, natural products have played an important role in the development of new sources to treat inflammatory diseases [15]. Historically, the screening of sources for natural products led to the discovery of clinical drugs currently used in the pharmacological therapy [16]. Nonetheless, since natural products do not have a standard composition and they may provide compounds with a unique structural activity there is interest in identifying the potential biological therapeutic in new plant extracts [17].

*Malva sylvestris* L. ethnopharmacological literature has been in wide use since ancient times for its emollient, wound healing, antioxidant, antimicrobial and anti-inflammatory properties [16]. Additionally, it has a long history of use as a food or condiment that can be prepared as simple additive in soups or salads [17]. Population studies show the incidence of consumption as a nutraceutical food is particularly due to the presence of fatty acids, which may help prevent several diseases and free radical scavenging activity, and protect against chronic diseases [18]. Recommendations that the plant’s biological activity be used to treat and prevent inflammation is found in the literature due to the controlling eicosanoid products, including the pro-inflammatory mediator prostaglandin and a variety of cytokine targets like IL-1β IL-6, IL-8 and granulocyte-macrophage colony-stimulating factor GM-CSF. Phytochemical identification shows that malvidin 3-glucoside, scopoletin and quercetin may be connected to the biological activity [19].

Due to its widespread and medicinal importance, the aim of this study was to investigate the extract and fractions of *M*. *sylvestris* in a bioguided phytochemical study for anti-inflammatory, anti-osteoclastogenic mechanisms and antioxidant effects.

## Material and Methods

### Ethics statement

Mice were inspected at least once a day and checked for feeding, bedding and all environmental aspects. Moreover, in the protocol three strategies were included to manage pain and distress: non-pharmacological approach, pharmacological interventions or euthanasia. The strategy chosen varied according to the special requirements of each experiment conducted (procedure performed, type of analgesia, degree, etc.) and followed a veterinarian’s instructions. None of the animals died prior to the experimental endpoint, but we considered a humane endpoint to minimize pain and distress. The euthanasia agent was carbon dioxide and a confirmatory cervical dislocation [20].

### Preparation of the extract and fractions

*M*. *sylvestris* (Malvaceae) leaves were purchased from a local farmer in the municipality of “Princesa Isabel”, Paraiba (07°44'12” S and 37°59'36" W). This plant is not an endangered or protected species and was registered in the herbarium of the University of Sao Paulo (USP), receiving an identification number (ESA voucher # 121403, Det.: Lopes, S.). The leaves were dried in a forced circulation oven (Marconi, Piracicaba, Brazil) for 24 h, at a 40°C, and then crushed in a grinder (30 mesh) to obtain an adequate granulometry. The extraction was conducted with absolute ethanol under climate control (25°C) and subjected to exhaustive extraction (7 days) in a rotary shaker (Labnet, 211DS) protected against light [19]. In the sequence, samples were filtered in a qualitative filter (80 g/m^2^), concentrated by evaporation (Buchi Rotavapor; Switzerland) and lyophilized (Christ, Osterode, Germany) for 72 h. The ethanol extract of *M*. *sylvestris* (MSE) was stored at -20°C. The MSE was re-suspended (1 g/L, in ethanol 70%) and the mobile solvent system selected according to the sequence: hexane, chloroform and ethyl acetate. At the end of three partitions, the hexane (HF), chloroform (CLF) and ethyl acetate fractions (EAF) were obtained and the residue was called the aqueous fraction (AF). Several extractions were conducted and monitored with thin-layer chromatography (TLC) with silica gel 60 (Merck^®^, F-254) used for the stationary phase. The plates were then conditioned on a glass-lid and flat-bottom chamber pre-saturated with an analytical grade mobile phase of hexane, acetic acid: ethanol (75: 25: 5). Anisaldehyde reagent (4-methoxybenzaldehyde, acetic acid, sulfuric acid, (1.0: 48.5: 0.5) was used for the developing process followed by heating in an oven at 100°C for two minutes [21].

### Pharmacological analysis

#### Anti-inflammatory analysis

***Animals*:** Male Balb/c albino mice (20–25g), SPF (Specific—pathogen—free), were purchased from CEMIB/UNICAMP (Multidisciplinary Center for Biological Research, SP, Brazil). The mice were kept in a temperature-controlled room (22 ± 2°C) for a 12 h light/12 h dark cycle, humidity 40–60%, with food (standard pellet diet) and water provided *ad libitum*. The experiments were conducted in accordance with the Guide for the Care and Use of Laboratory Animals and had received prior approval from the local Animal Ethics Committee (Comissão de Ética no Uso de Animais (CEUA), Ethics Committee on Animal Use/UNICAMP, process number 2790–1).

***Neutrophil migration in the peritoneal cavity***: To determine the anti-inflammatory activity of MSE and fractions, the neutrophil migration test was assessed. The animals were randomly assigned into groups. MSE and the HF, CLF, EAF and AF groups were administered by oral gavage at concentrations of 3, 10 and 30 mg/kg. The vehicle control group received (saline/ethanol 1%) and the positive control dexamethasone (Decadron^®^) at 1 mg/kg subcutaneously. Regarding the saline group, all the groups were challenge by intraperitoneal injection of carrageenan at 500 μg/mL in naive mice. The mice were euthanized 4 h after the challenge with carrageenan. The abdomens were gently massaged and the peritoneal cavity cells were harvested by washing the cavity with 3 mL of phosphate-buffered saline (PBS) containing EDTA (1 mM). A blood-free cell suspension was carefully aspirated with a syringe. The volumes recovered were similar in all experimental groups and equal to approximately 95% of the injected volume. Aliquots of peritoneal washing were stored in a freezer at –80°C for subsequent cytokine dosage. The total counts were performed in a Neubauer chamber. The differential cell count included 100 cells in total presented in smears conducted using a cytocentrifuge (Cytospin 3; Shandon Lipshaw) and followed by panoptic staining. Cells were counted using an optical microscope (Carl Zeiss, 40x) and the results were presented as the number of neutrophils per milliliter [22].

***Carrageenan-induced paw edema***: A paw edema was induced by subplantar injection of 0.05 mL of lambda carrageenan (1% w/v in 0.9% of saline) into the left hind paw of the mice. An equal volume of vehicle was injected into the contralateral paw. The volume of both hind paws up to the ankle joint was measured with a plethysmometer (UGO Basile, Model 7140) immediately before the 0, 1, 2, 3, 4 and 5 h post-carrageenan. The difference in the volumes between the hind paws was a measure of the edema (mL). The MSE and the bioactive fraction previously selected in the neutrophil migration model were administered by oral treatment (30 mg/kg). The reference drug indomethacin (10 mg/kg) was given intraperitoneally. The control (saline/ethanol 1%) were given orally 1 h before the subplantar injection of the phlogistic agent [23*]*.

***Cytokine quantification***: Based on a previous test (neutrophil migration assay), the MSE and the bioactive AF were selected for the quantification of proinflammatory cytokines. The mice were treated orally with the MSE or the AF (30 mg/kg) 1h before the administration of inflammatory stimulation by intraperitoneal (i.p.) injection of carrageenan at 500 μg/mL. After 4 h, the animals were euthanized and the exudate fluid was collected from peritoneal cavities and homogenized by using a vortex in a 500 μL of the appropriate buffer containing protease inhibitors (Sigma, St. Louis, MO, USA). TNF-α and IL-1β levels of both experiments were determined by ELISA using protocols supplied by the manufacturers (Peprotech, Rocky Hill, NJ, USA).

#### Osteoclastogenic assays

***Cell culture***: RAW 264.7 cells were purchased from the Rio de Janeiro cell bank (BCRJ code: 0212; Rio de Janeiro, RJ, Brazil) and cultured in Dulbecco’s Modified Eagle’s Medium (Life Technologies, Carlsbad, CA, USA). The culture medium contained phenol red and L-glutamine supplemented with 10% (v/v) of serum fetal bovine (Life Technologies, Carlsbad, CA, USA), penicillin/streptomycin (100 U/mL, Invitrogen Life Technologies, CA) and cultured in a T75 flask (Corning Inc., New York, NY, USA). Cells were maintained in a humidified incubator at 37°C in 5% CO_2_ and subcultured once a week at a 1: 20 ratio following harvesting with a cell scraper and receiving a fresh new medium [9*]*.

***Cell viability***: A cell-based assay to screen the MSE and fractions (HF, CLF, EAF, AF) to measure the enzyme activity as a marker of viable cells. RAW 264.7 cells were seeded (~ 1x10^5^ cells/mL) in a 96-well plate and incubated for 24 h at 37°C with 5% CO_2_. The MSE and fractions were first diluted in ethanol 1% and then (0.1–1000 μg/mL) added to the cell culture serially and incubated for 24 h. After incubation, the supernatant was discarded and the cells were washed with PBS. Fresh medium solution without phenol red and with 3-(4,5-dimethylthiazol-2-yl)-2,5-diphenyltetrazolium (MTT) 0.5 mg/mL was then incubated (10 μL per 100 μL medium) for an additional 4 h. Afterward, the cell growth medium was replaced by 0.04 N HCl in isopropanol and colorimetric measurements were performed with a microplate reader (Asys HiTech GmbH, Cambridge, United Kingdom) at 570 nm [24].

***Analysis of gene expression***: Quantitative PCR (qPCR) was performed to evaluate the possible effects of MSE and fractions on the expression of predominant osteoclast marker genes. In addition, it was evaluated whether the natural products could control the transcription of the genes involved in bone metabolism. The genes analyzed were: carbonic anhydrase II (*CAII*), cathepsin K (*Ctsk*), tartrate-resistant acid phosphatase (*TRAP*) and glycerol 3 phosphate dehydrogenase (*GAPDH*). The primer sequences were: *CAII* (forward: TGGTTCACTGGAACACCAA, reverse: CACGCTTCCCCTTTGTTTTA), *Ctk* (forward: CAGCTTCCCCAAGATGTGAT, reverse: AGCACCAACGAGAGGAGAAA), *TRAP* (forward: CCCTCTGCAACTCTGGACTC, reverse: TAGAGGCGAACAGGAAGGAA), *GAPDH* (forward: AACTTTGGCATTGTGGAAGG, reverse: ACACATTGGGGGTAGGAACA). RAW 264.7 cells (*~* 1x10^6^ cells/mL) were seeded in 24-well plates for 24 h and treated with a 10 μg/mL concentration of the MSE and fractions in serum-free medium for 24 h. The stimulatory response was induced by 1 μg/mL LPS (*Escherichia coli*, Sigma Aldrich, St. Louis, Mo). Cultures were washed twice with PBS and RNA was subsequently isolated using the RNeasy Mini Kit (Qiagen, Valencia, CA, USA) following the manufacturer’s protocols. RNA was treated with the DNase Set (Qiagen, Valencia, CA, USA). The cDNA was synthesized from total RNA using the SuperScript^®^ III First-Strand Synthesis System and random primers (Invitrogen, Carlsbad, CA, USA). The relative transcript amounts were quantified by qPCR with 10 ng of each cDNA and SYBR Green PCR Master Mix (Applied Biosystems, Foster City, CA, USA). The reactions were performed in the instrument StepOnePlus^™^ (Applied Biosystems, Foster City, CA, USA). GAPDH was used as an endogenous control. The relative gene copy number was calculated using the 2^ΔΔ^CT method [25].

***TRAP staining***: To examine the effect of the MSE and fractions on sRANKL-induced osteoclastogenesis in RAW 264.7 macrophage cells, a quantitative measurement was conducted. Osteoclast formation was measured by the quantification of TRAP+ multinucleated osteoclasts per well, using light microscopy. RAW 264.7 cells were seeded in 96-well plates (~5x10^3^ cells/mL), stimulatory response was induced by sRANKL (50 ng/mL) and treated with the MSE and fractions at 10 μg/mL, and the cell culture medium was α-MEM (Sigma Aldrich, St. Louis, MO, USA) with 10% fetal bovine serum (Life Technologies, Carlsbad, CA, USA). After 6 days, cells were fixed with 4% paraformaldehyde, washed with PBS, and stained for TRAP (Sigma Aldrich, St. Louis, MO, USA). TRAP-positive multinucleated (>3 nuclei) cells were counted as osteoclast-like cells [9].

#### Gelatin zymography

RAW 264.7 cells (*~* 1x10^6^ cells/mL) were seeded into 24-well plates for 24 h. Inflammatory response was induced by 1 μg/mL LPS for 48 h (Sigma Aldrich, St. Louis, Mo). The test concentrations of the MSE and the HF, CLF, EAF and AF were 10 μg/mL. The supernatant was collected and the amount of total protein was measured using the Pierce BCA Protein Assay Kit (Thermo Scientific, Rockford, IL, USA). An equal amount of protein was designed by electrophoresis in Tris-Glycine Gel (Novex^®^, Life Technologies, Carlsbad, CA) under non-reducing conditions. The protein separated in the gel was developed using manufacturer-supplied developing buffer (Novex^®^, Life Technologies, Carlsbad, CA). Subsequently, the developed gelatin gel was stained with Coomassie R-250 stain [9].

#### Antioxidant assays

***DPPH*:** The chemical reactions comprised in 0.5 mL of MSE and fractions, 3.0 mL of absolute ethanol, and 0.3 mL of DPPH radical in a 0.5 mM ethanol solution, at room temperature incubation for 45 min, and the activity quantified in μmol Trolox/g of sample dry weight. The calibration curve was generated using a concentration range of the standard compound Trolox. Several MSE and fraction concentrations were used, and readings were monitored at 517 nm using a spectrophotometer (Shimadzu, Japan). The antioxidant activity measured by the DPPH free radical method can be expressed as IC_50_, i.e., the antioxidant concentration required to reduce the initial DPPH radical by 50%. The sample concentration required the initial DPPH radical by 50% [26].

***ABTS•+*:** The antioxidant activity by the ABTS•+ method (2,2′-azinobis-3-ethylbenzothiazoline-6-sulfonic acid) was assessed according to the method described by Re et al. (1999) [27] with modifications. The ABTS radical was formed by the reaction of 7 mM ABTS•+ solution with 140 mM potassium persulfate solution, incubated at 25°C in the dark for 12–16 h. Once formed, the radical was diluted with ethanol (analytical grade) to an absorbance of 0.700 ± 0.020 at 734 nm. Three different dilutions of each of the samples were prepared independently in triplicate. After that, 30 μL of the MSE and fraction dilution were transferred to test tubes with 3.0 mL of ABTS radical in the dark. The absorbance was read at 734 nm after 6 min of reaction using ethanol as a blank. Trolox, a synthetic water-soluble antioxidant analog of vitamin E, was used as the reference at concentrations ranging from 100 to 2000 μM, and the results were expressed as μM Trolox/g sample.

### Chemical analysis

#### HPLC analysis

A Shimadzu SCL-10Avp system equipped with photodiode array detector (SPD-M10Avp, Shimadzu Co., Kyoto, Japan), a SIL-10AF auto injector and LC-6AD pumps were used to perform the high-performance liquid chromatography (HPLC) analysis. For the analytical test, diluted AF solution at 1.1% in methanol/water (80/20, v/v) was filtered (Millipore– 0.22 μm) and aliquots of 20 μL were injected into an ODS-A column (4.6 mm x 250 mm, 5 μm) maintained at a constant temperature of 28°C. The mobile phase consisted of water/formic acid (99.9/ 0.1, v/v) (solvent A) and acetonitrile/formic acid (99.9/ 0.1) (solvent B) at a constant flow rate of 1.0 mL/min. The gradient started with 5% B and increased to 7% B (7 min), 20% B (50 min), 45% B (70 min), 100% B (85 min), held at 100% B for 10 min, and decreased to 5% in 100 min. The chromatograms were analyzed using Class-VP^®^ software; in addition, authentic standards of phenolic acids (gallic acid, *p*-coumaric acid, ferulic acid, caffeic acid, syringic acid, sinapic acid, vanillic acid) and flavonoids (quercetin, rutin, quercetin-3-β-D-glucoside, procyanidin B1, procyanidin B2, catechin and epicatechin) (Sigma-Aldrich, St. Louis, MO, USA) were examined [26].

#### Mass spectrometric analysis of the bioactive fraction

The tandem mass spectrometry (MS/MS) system employed to confirm analyte identities was a Quattro LC triple quadrupole (Micromass, Manchester, UK) fitted with a Z-electrospray (ESI) interface operating in negative ion mode. The temperatures of source block and desolvation gas were set at 100°C and 450°C, respectively. Nitrogen was used as both desolvation (nearly 380 L/h) and nebulizer (nearly 38 L/h) gas, while argon was used as the collision gas. The voltages employed in the ESI source during the analyses were 40 V for the cone, 3 kV for the capillary and 3 V for the extractor. For rutin identity confirmation, the analyses were carried out in multiple-reaction monitoring (MRM) mode using collision energies of 15–25 eV. ESI positive and negative modes were employed. For ESI positive mode, protonated ions [M+H]^+^ (m/z 611) and sodiated ion [M+Na]^+^ (m/z 633) as well as their respective product ions were monitored. For ESI negative mode, deprotonated ion [M-H]^-^ (m/z 609) and its respective product ions were monitored. For this analysis, 23.8 mg of the AF were diluted with 1 mL MeOH: 0.1% formic acid (1:1, v/v) and injected into the LC-MS/MS system under a flow of 20 μL min^-1^. Table 1 presents the ion transitions under MRM mode employed to monitor for rutin in the sample.

**Table 1:** Ion transitions under MRM mode employed to monitor for rutin in the sample.

Compound	Ionization mode	Molecular ion	Fragments/Productions
		(*m/z*)*	
	Positive	611 [M+H]^+^	464, 303, 147, 129
Rutin	Positive	633 [M–H]^-^	486, 331, 324, 133
	Negative	609 [M+Na]^+^	301, 271, 179, 151

*mass number/charge number

### Statistical analysis

Continuous variables are presented as mean values ± SD. The Shapiro-Wilk test was used for the assessment of normality. All reported p-values are compared to a significance level of 0.05. For multiple group comparisons, the data were subjected to a one-way analysis of variance (ANOVA). Means and Tukey's significant difference for pair-wise differences in group comparisons were used to determine overall difference between the groups. The Bonferroni post-test indicated a significant difference between the controls. Data were analyzed using STATA^™^ (version 10.0, Stata Corporation, College Station, TX, USA) and GraphPad (version 5.*0*, GraphPad Software Inc., San Diego, CA).

## Results

### Pharmacological analysis

#### Neutrophil migration in the peritoneal cavity

The MSE and fractions were primarily tested for anti-inflammatory effects using a neutrophil migration model as an *in vivo* model to screen for anti-inflammatory compounds. The results showed that the oral administration of 30 mg/kg of the MSE decreased the influx of neutrophils into the peritoneal cavity compared to the carrageenan group (ρ<0.05). The HF fraction did not present any biological activity in all doses tested (ρ>0.05). The CLF and AF groups had no statistical differences between the 3, 10 and 30 mg/kg doses. For the EAF group the highest dose of 30 mg/kg was more efficient than lowest one 3 mg/kg (ρ<0.05).

**Fig 1:**
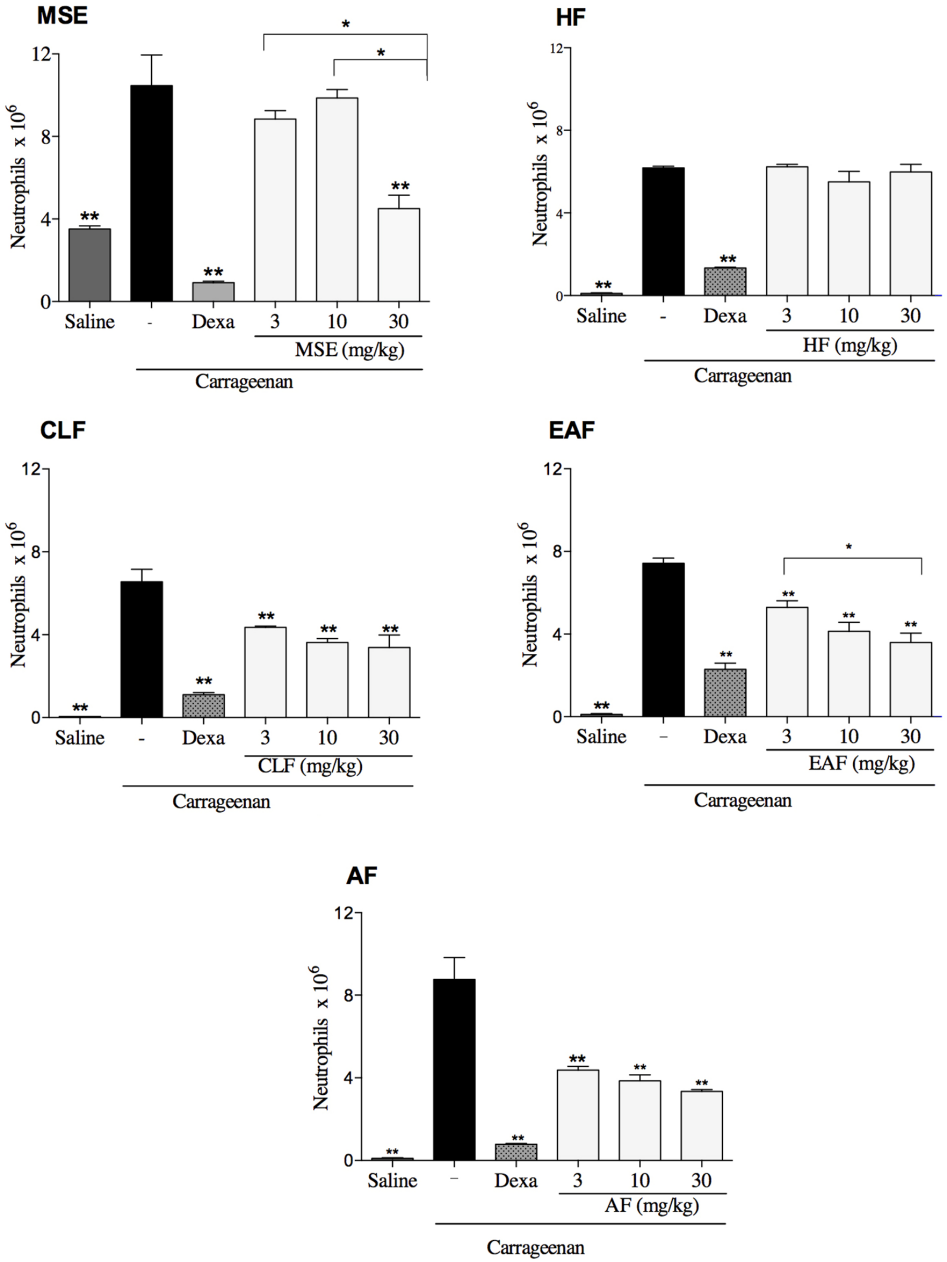
Inhibitory effect of the MSE, HF, CLF, EAF, AF on neutrophil migration into the peritoneal cavity induced by carrageenan. Neutrophil migration was determined 4 h after the injection of carrageenan 500 μg/cavity. Mice previously treated with the vehicle, MSE (ethanol extract of *M*. *sylvestris*), HF (hexane fraction), CLF (chloroform fraction), EAF (ethyl acetate fraction) and AF (aqueous fraction). The data are expressed as mean ± SD, n = 6. Symbols indicate statistical difference (p < 0.05, ANOVA, Tukey’s post-test) ** compared to the carrageenan group; * comparison between the concentrations tested of 3, 10 and 30 mg/kg.

The fractionation method used for the MSE was effective, showing that the CLF, EAF and AF concentrated the biological activity and reduced the neutrophil influx in all doses tested. However, the AF yielded the best results, showing a significant reduction of neutrophil migration in all doses tested and the lowest percentages of inflammatory cells compared to the other fractions (Fig 1).

#### Carrageenan-induced paw edema

The MSE and the bioactive AF were verified for antiedematogenic activity. The results for the AF demonstrated a biological activity in the first hour analyzed. AF was comparable to the positive control indomethacin for the second and third hours analyzed as shown in (Table 2).

**Table 2:** Effect of MSE and the AF on carrageenan-induced paw edema on mice.

Treatment (mg/kg)	Mean edema ΔV mL percent inhibition				
	1	2	3	4	5
Control	0.08±0.02	0.13±0.02	0.17±0.01	0.12±0.02	0.12±0.02
Indomethacin 10	0.05±0.03 (37.5%)	0.05±0.03* (61.5%)	0.08±0.02* (52.9%)	0.08±0.02* (33.3%)	0.08±0.02* (33.3%)
MSE 30**	0.06±0.02 (25%)	0.08±0.02* (38.4%)	0.07±0.01* (58.8%)	0.11±0.03 (8.3%)	0.11±0.03 (8.3%)
AF 30***	0.04±0.01* (50%)	0.06±0.02* (53.8%)	0.07±0.02* (58.8%)	0.10±0.02 (16.6%)	0.09±0.02 (25%)

All the data are expressed as mean ± SD, n = 6.

*Symbol indicate statistical difference (p < 0.05, ANOVA, Bonferroni post-test) and compared to the control) group.

**MSE: *M*. *sylvestris* extract,

***AF: aqueous fraction.

#### Cytokine assay

**Fig 2:**
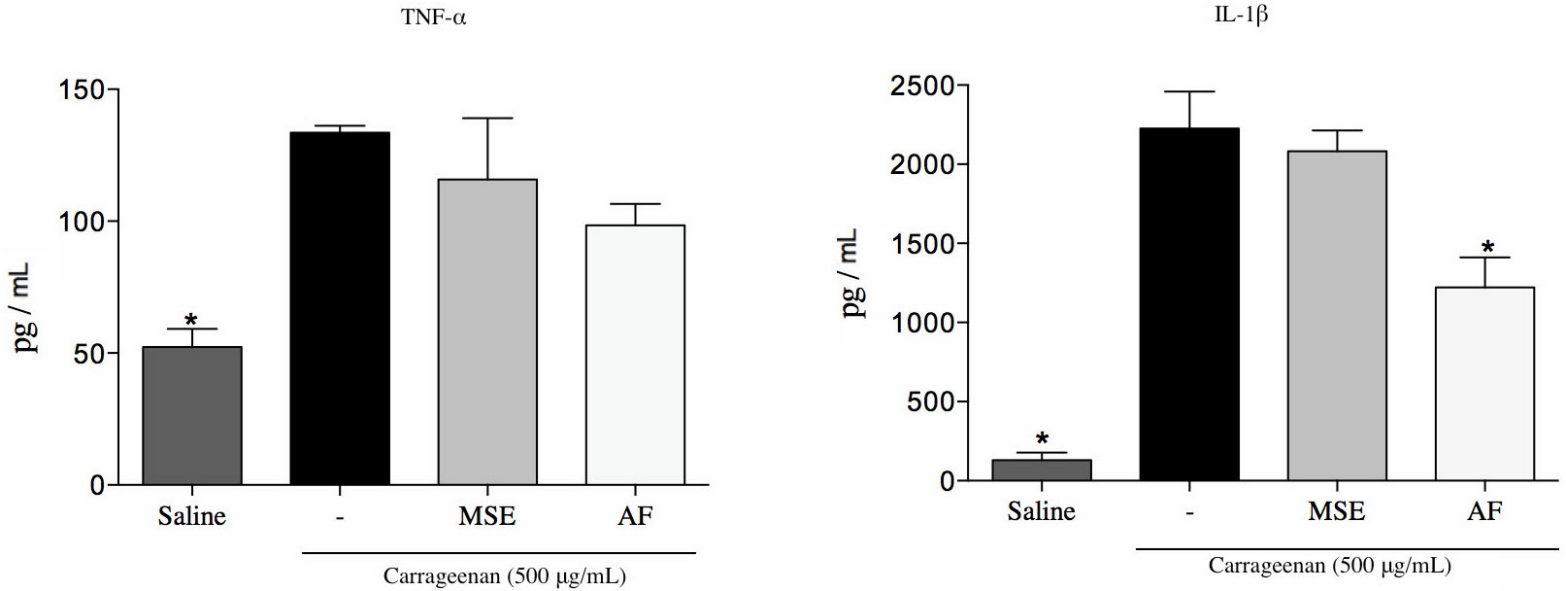
Quantification of TNF-α and IL-1β in the peritoneal cavity. Mice were previously treated with the vehicle, the MSE and the AF at a dose of 30 mg/kg 1h before the carrageenan injection. Data are expressed as mean±SD, n = 6. Symbols indicate statistical difference (p<0.05, Dunnett’s test); *p<0.05 compared to the carrageenan group.

The administration of the AF at a dose of 30 mg/kg significantly reduced the level of cytokine IL-1β (54%) compared to the control group vehicle (p<0.05). Thus, non-significant differences were found at TNF-α expression levels (Fig 2).

#### Cell viability assay

**Fig 3:**
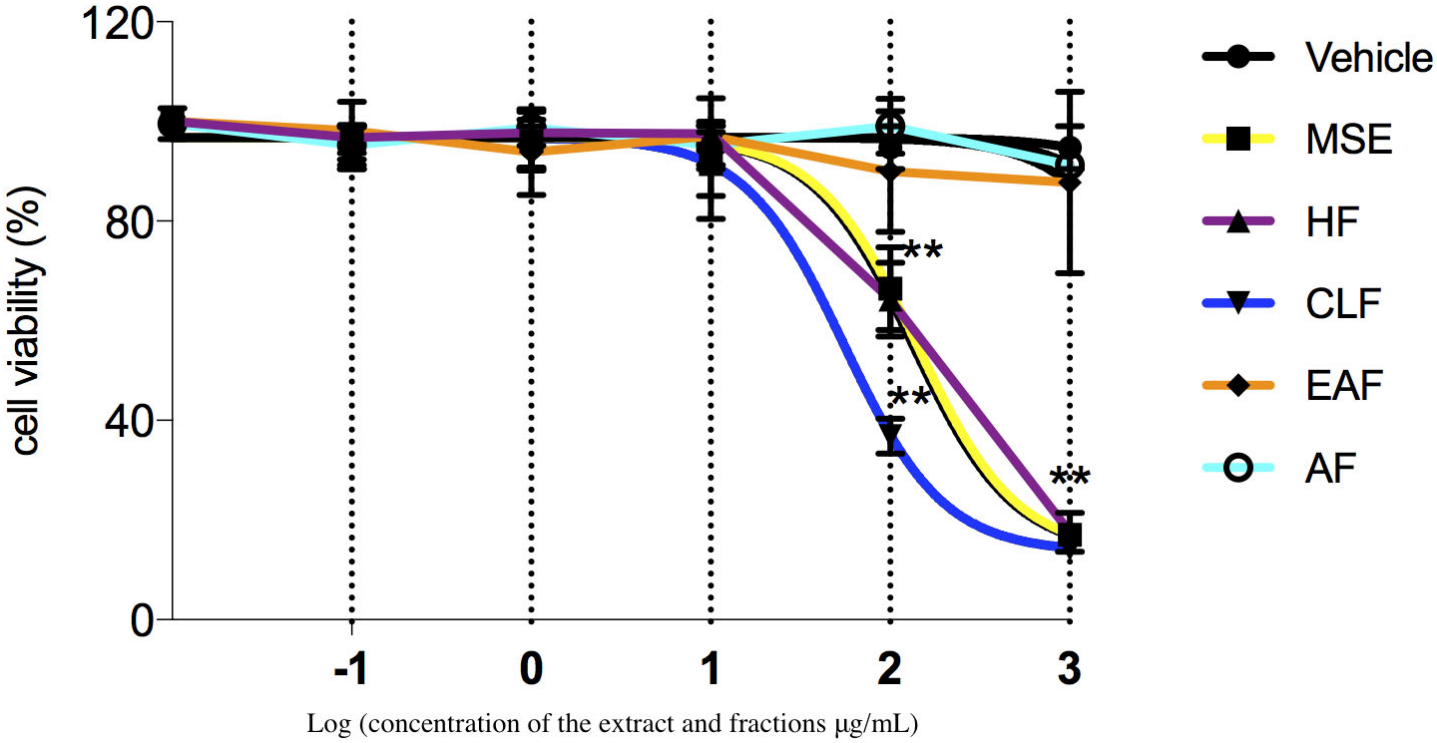
Effect of MSE and fractions on cell viability. Log dose response of MSE, HF, EAF, and AF on RAW 264.7 cells. Significant difference between fractions at the indicated dose (p<0.05). **Significant difference between IC50 values (p < 0.05). Data were expressed as mean±SD, n = 9.

The viability test showed a concentration-dependent effect, where the MSE, HF, and CLF at concentrations higher than 10 μg/mL were toxic for the cells. The AF and EAF did not affect the cell viability at any of the concentrations tested when compared to the control group (non-treated) (p>0.05) (Fig 3).

#### Analysis of gene expression

**Fig 4:**
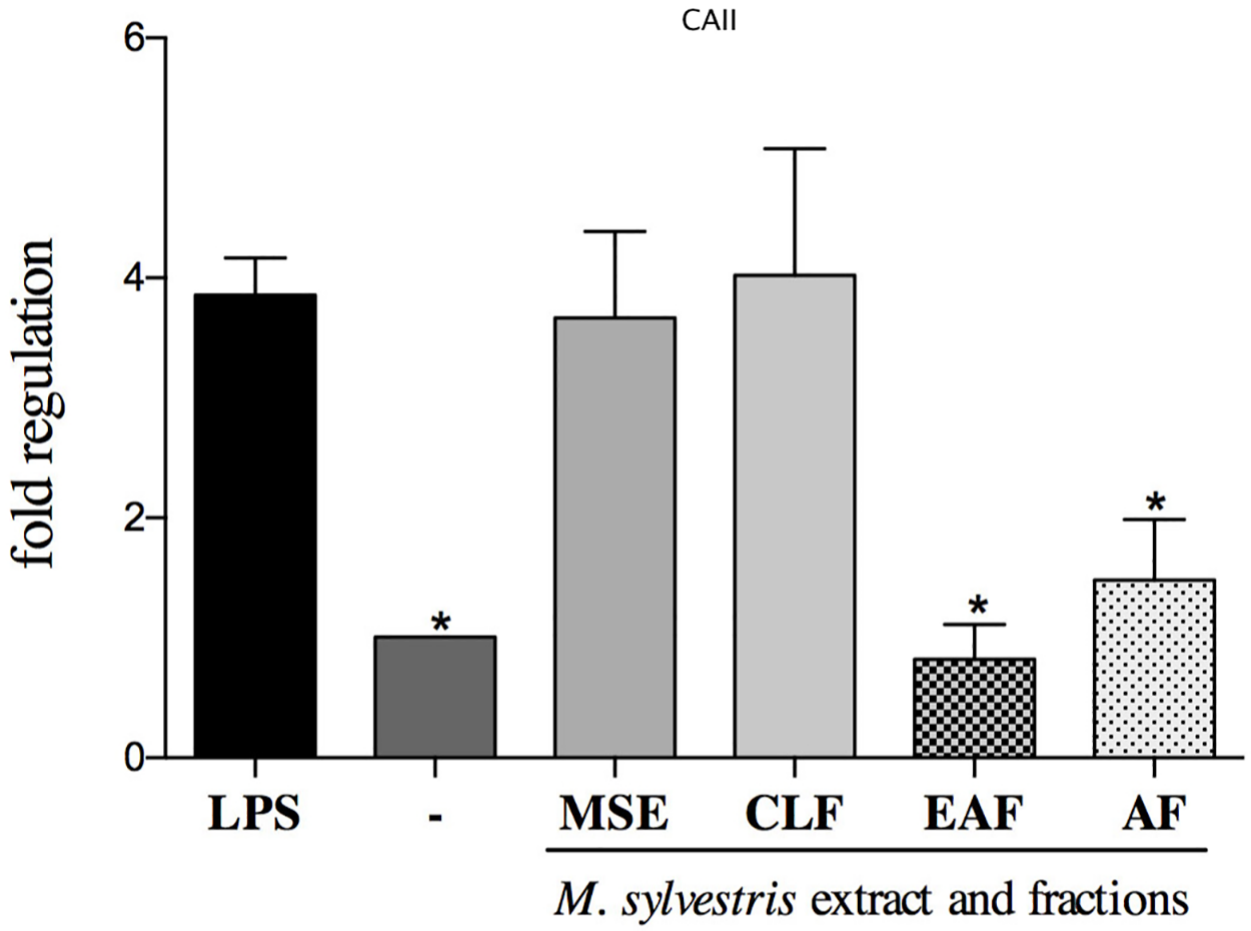
Effect of MSE and fractions on *CAII* (carbonic anhydrase II) expression levels. Quantification of the relative transcript amounts performed by qpcR with 10 ng of each cDNA. Data quantification was performed using 2(-DeltaDeltaC(T)). Statistical analyses were performed by one-way ANOVA followed by Dunnett’s post-hoc tests. *p<0.05 significantly different from LPS-stimulated cells.

**Fig 5:**
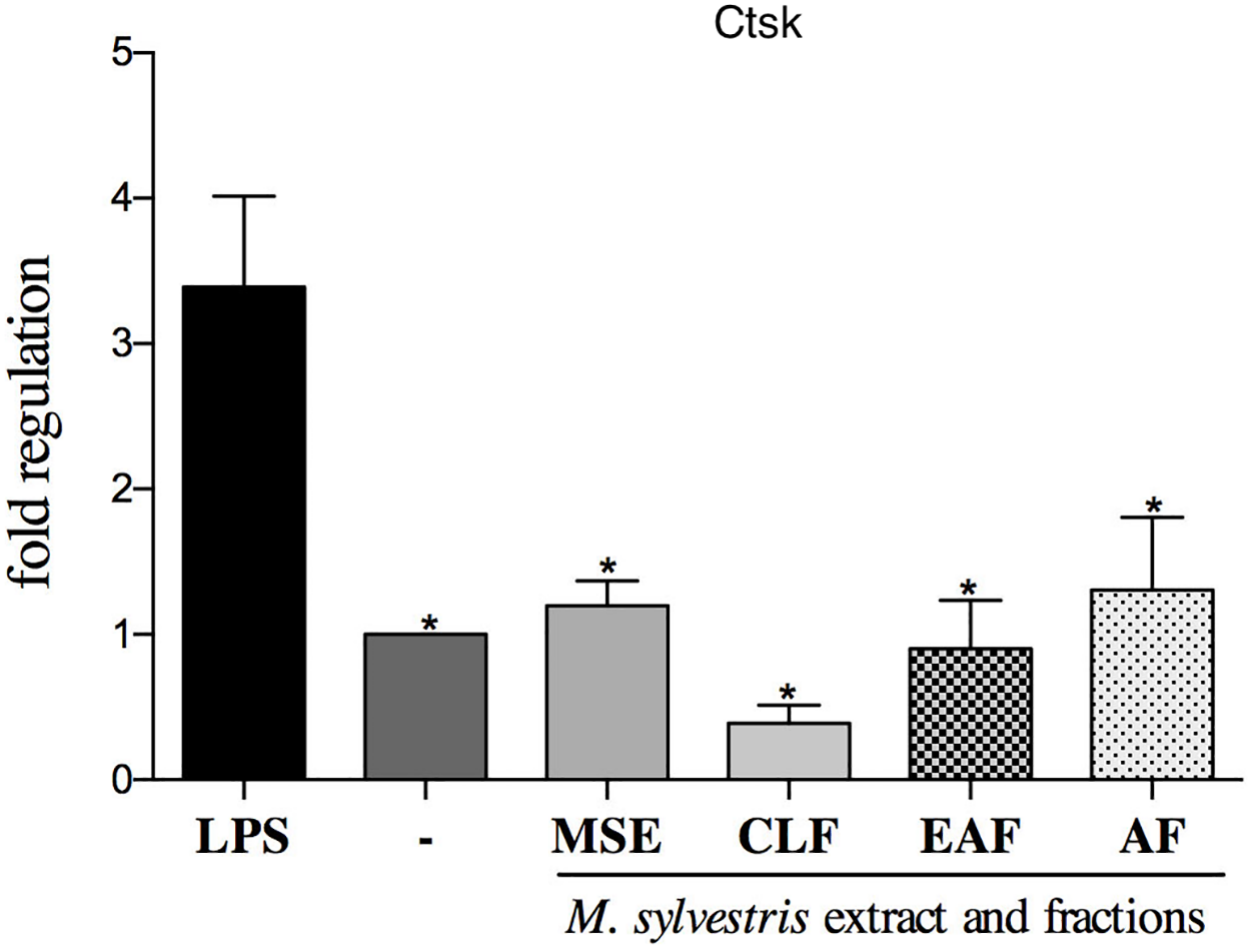
Effect of MSE and fractions on *Ctsk* (cathepsin K) expression levels. Quantification of the relative transcript amounts performed by qpcR with 10 ng of each cDNA. Data quantification was performed using 2(-DeltaDeltaC(T)). Statistical analyses were performed by one-way ANOVA followed by Dunnett’s post-hoc tests. *p<0.05 significantly different from LPS-stimulated cells.

**Fig 6:**
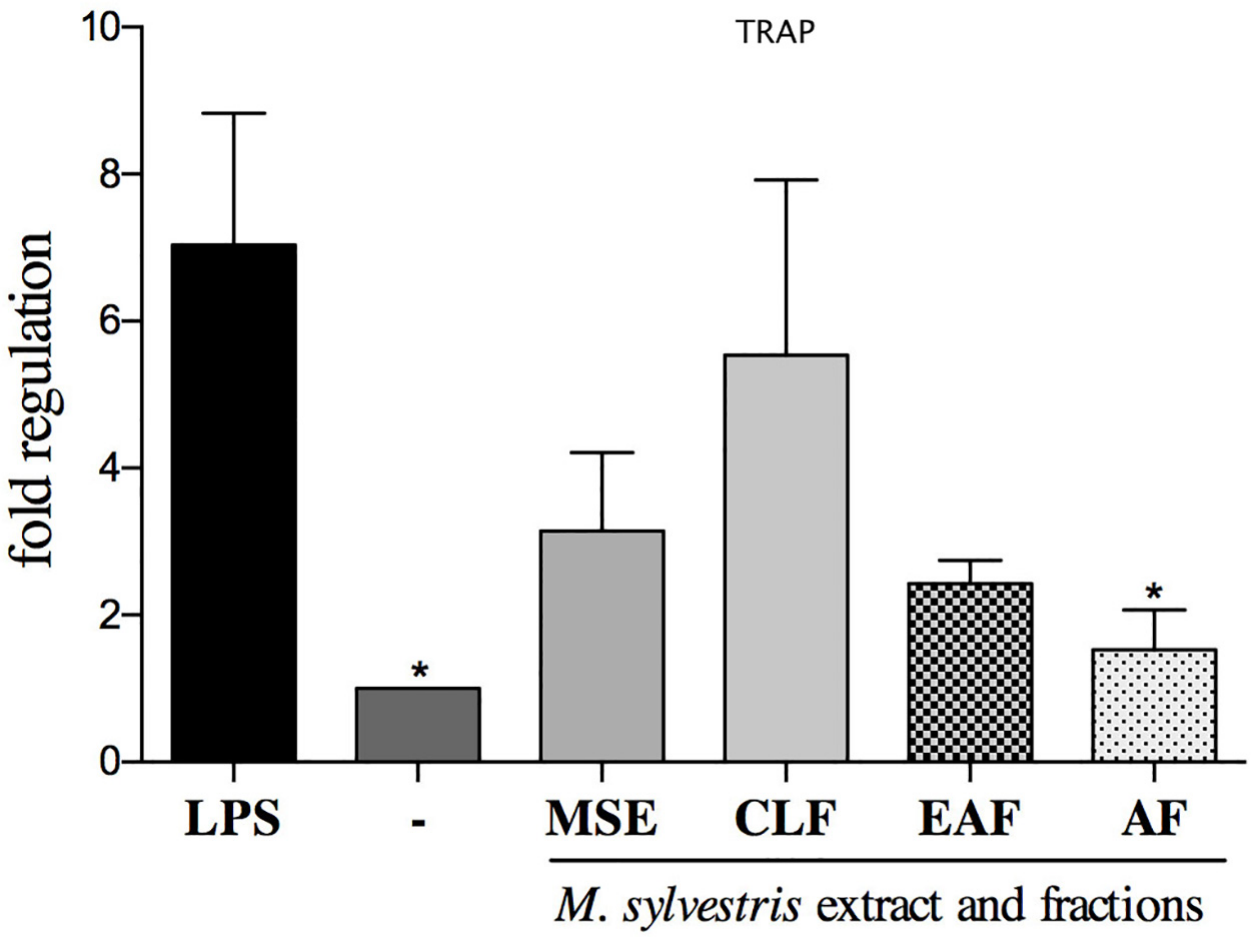
MSE and fractions effect on *TRAP* (tartrate-resistant acid phosphatase) expression levels. Quantification of the relative transcript amounts performed by qpcR with 10 ng of each cDNA. Data quantification was performed using 2(-DeltaDeltaC(T)). Statistical analyses were performed by one-way ANOVA followed by Dunnett’s post-hoc tests. *p < 0.05 significantly different from LPS-stimulated cells.

Gene expression analysis showed that the AF was the only treatment that had the ability to downregulate all the study genes: *CAII*, *Ctsk* and *TRAP* (p<0.05) (Figs 4, 5 and 6). The EAF and AF fractions had the ability to suppress the transcription of *CAII* compared to the control group with LPS (p<0.05) (Fig 4).

The gene transcription of Ctsk was controlled by the treatments with the AF and EAF (p>0.05) (Fig 5).

The MSE, CLF, EAF and AF downregulated the gene expression of TRAP (p>0.05) (Fig 6).

#### Tartare-resistant acid phosphatase activity assay

**Fig 7:**
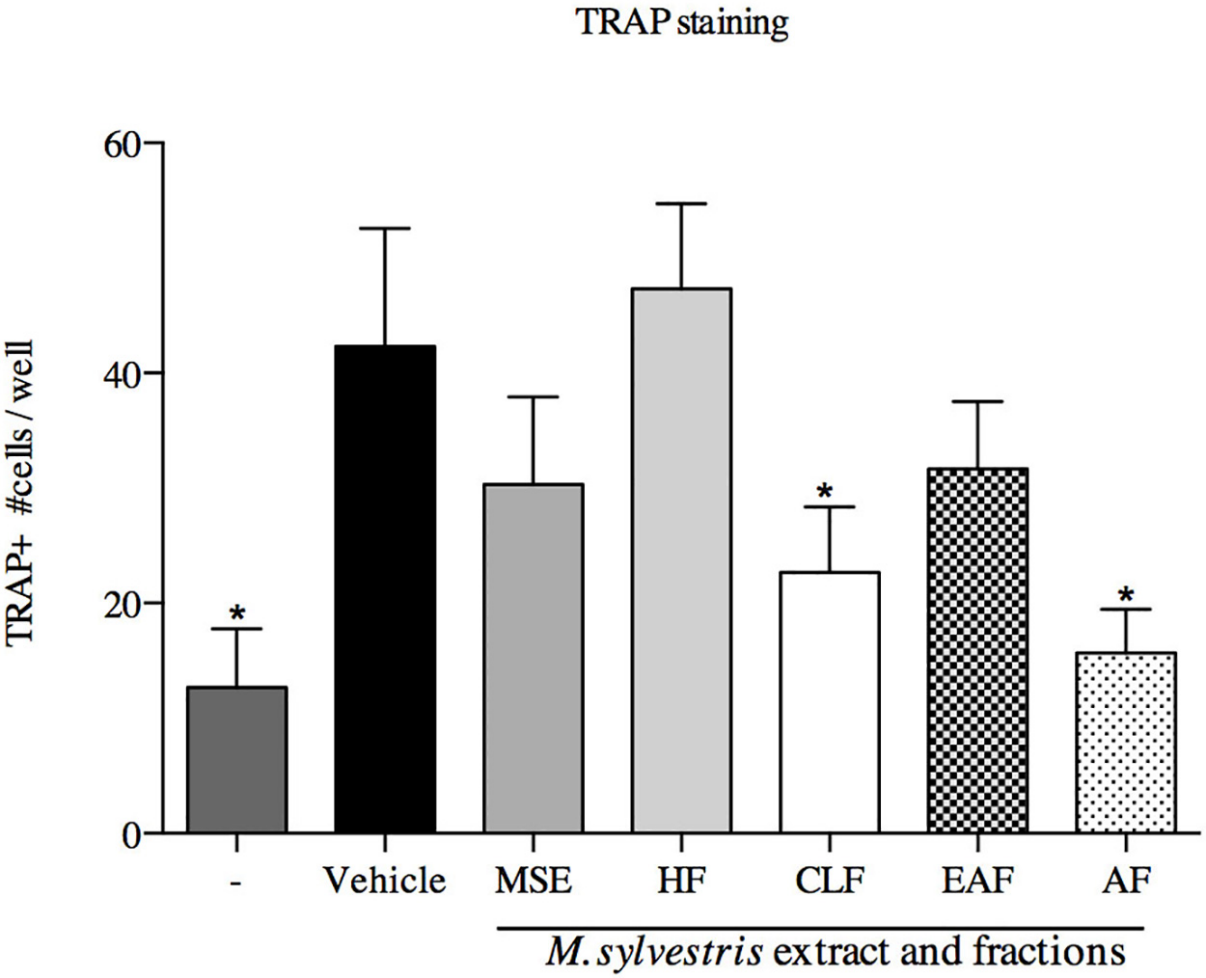
Activity of *M*. *sylvestris* and fractions in RANKL-mediated osteoclast differentiation. RAW 264.7 cells were stimulated with sRANKL (50 ng/ml) for 6 days. Cells were fixed and stained for TRAP. TRAP+ multinuclear cells were counted. Data represent mean ± SD of three cultures. * p<0.05; significantly lower than sRANKL-stimulated group.

The number of TRAP-positive cells showed high expression levels in the stimulated control group (p<0.05). The CLF and AF treatments had the ability to reduce the osteoclastogenesis in RAW 264.7 cell lines (p<0.05) (Fig 7).

#### Gelatin zymography

**Fig 8:**
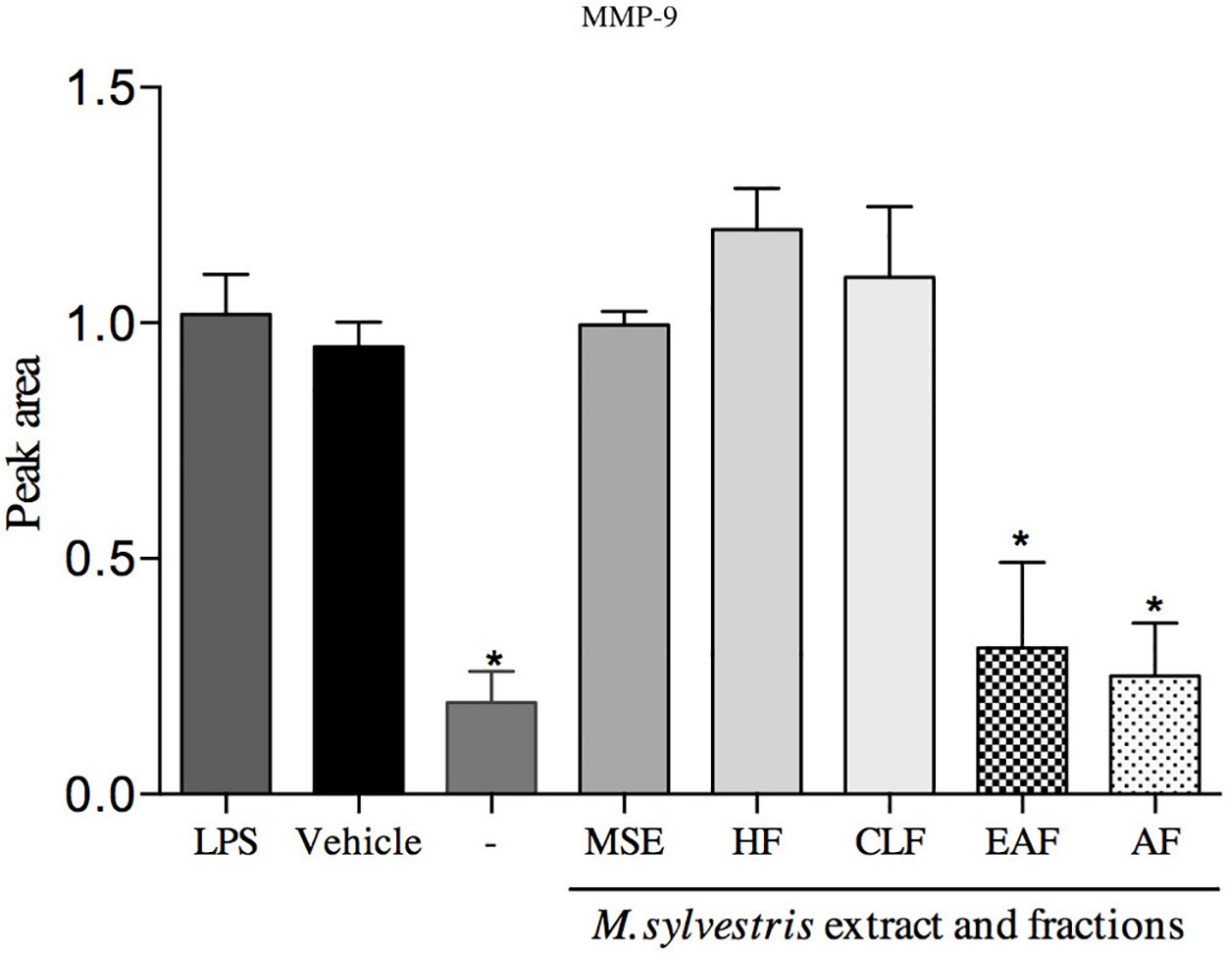
Effect of MSE and fractions on MMP-9 expression levels. Proteolysis activity was stimulated by LPS *(E*. *coli*) 1 μg/mL. Supernatant was mixed 1:1 with sample buffer and then applied to the gels. Quantification was performed by peak area and normalized by the protein ladder band. Data quantification was performed using ImageJ software. Statistical analyses were performed by one-way ANOVA followed by Dunnett’s post-hoc test. *p<0.05 significantly different from LPS-stimulated cells.

The results showed that two different fractions caused the reduction of gelatinolytic activity: the AF and EAF at the 10 μg/mL concentration. The non-treated group and the AF, EAF were not significantly different (p>0.05). In this study, MMP-9 activity decreased 69% and 75% (AF and EAF, respectively). In contrast, HF and CLF increased the enzyme activity (Fig 8).

#### DDPH and ABTS^•+^ assay

The results are presented in Table 3 and show that when the ABTS•+ method was used, the AF and EAF had the highest antioxidant activity (1.3 μmol Trolox/g and 1.1 μmol Trolox/g, respectively).

**Table 3:** Antioxidant activity of MSE and fractions using ABTS^*•+*^ and DPPH method.

Antioxidant activity		
Groups	IC_50_ DPPH (mg/mL)	ABTS^•+^ (μmol Trolox/g)
MSE	2.62±0.08^a^	0.34±0.03^a^
HF	6.01±1.78^b^	0.25±0.02^a^
CLF	1.78±0.20^c^	0.64±0.10^a^
EAF	0.94±0.04^d^	1.10±0.20^b^
AF	1.01±0.06^d^	1.30±0.20^b^

Reference values: BHT (0.1 mg/mL)^__^ 1268± 15 μmol Trolox/g (ABTS^•+^), (184 ± 2.6 μmol Trolox/g (DPPH). Averages of triplicates ± SD/means followed by letters showing the columns that differ statistically (p<0.05). Tukey's multiple comparison test. DPPH: 2,2-diphenyl-1-picryl-hydrazine; ABTS: 2,2′-azino-bis-3-ethylbenzothiazoline-6-sulfonic acid, BHT (butilhidroxitolueno).

Based on the DPPH method results, the samples where the concentration reduced the initial amount of DPPH radical by 50% were the EAF (0.94 g/L), followed by the AF (1.01 g/L), and the CLF (1.78 g/L). The lowest IC_50_ values can be considered good results in terms of antioxidant activity, given that a low concentration is required to reduce the DPPH free radical by 50%.

### Chemical analysis

#### HPLC analysis

**Fig 9:**
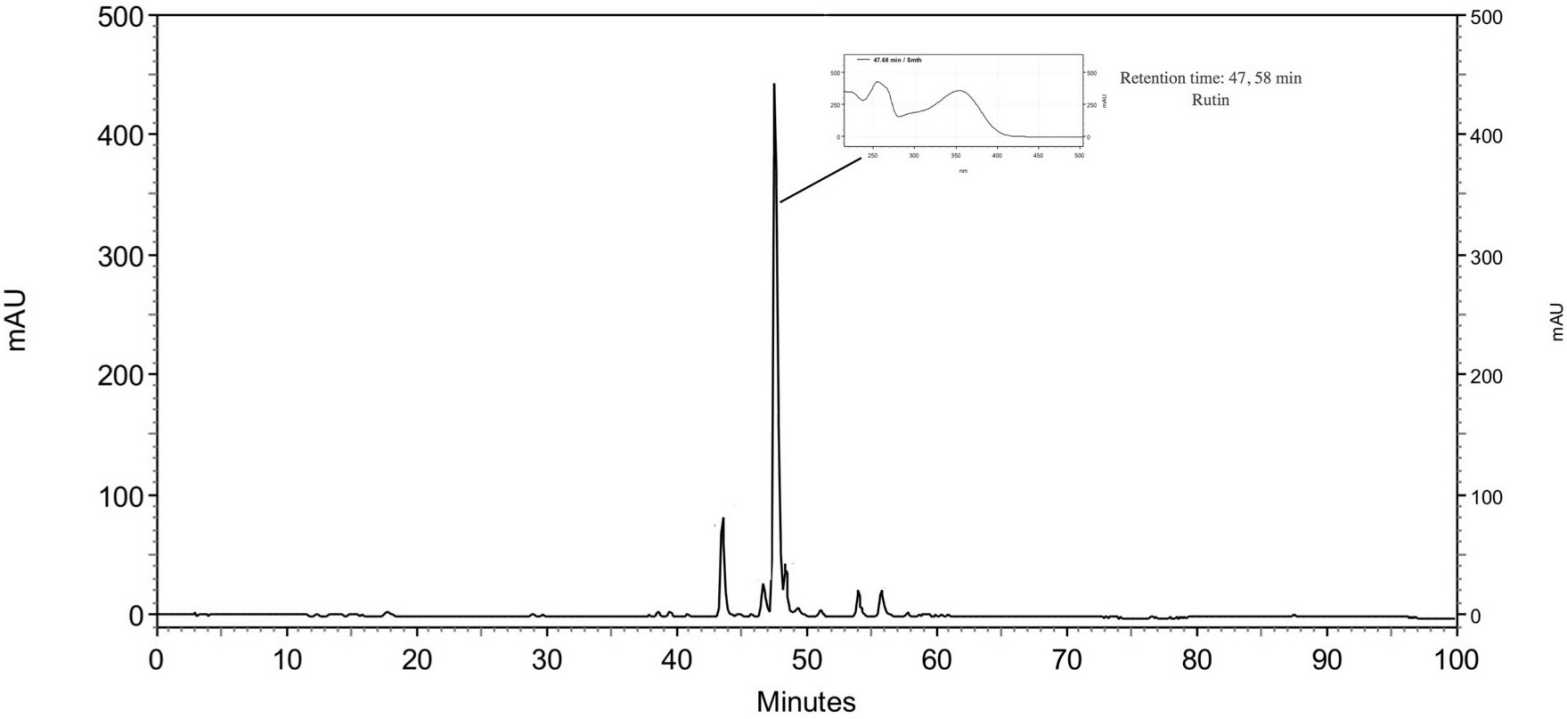
Analytical HPLC-PAD chromatogram of the AF of the ethanol extract of *M*. *sylvestris* recorded at UV 350 nm.

The HPLC analysis was used for the bioassay-guided fractionation and the major compound of the AF was identified and represented (Fig 9). The peak corresponding to rutin was identified at retention time 47.58 min and UV absorbance at 350 nm.

#### Mass Spectrometric Analysis of Bioactive Fraction (Aqueous Fraction)

**Fig 10:**
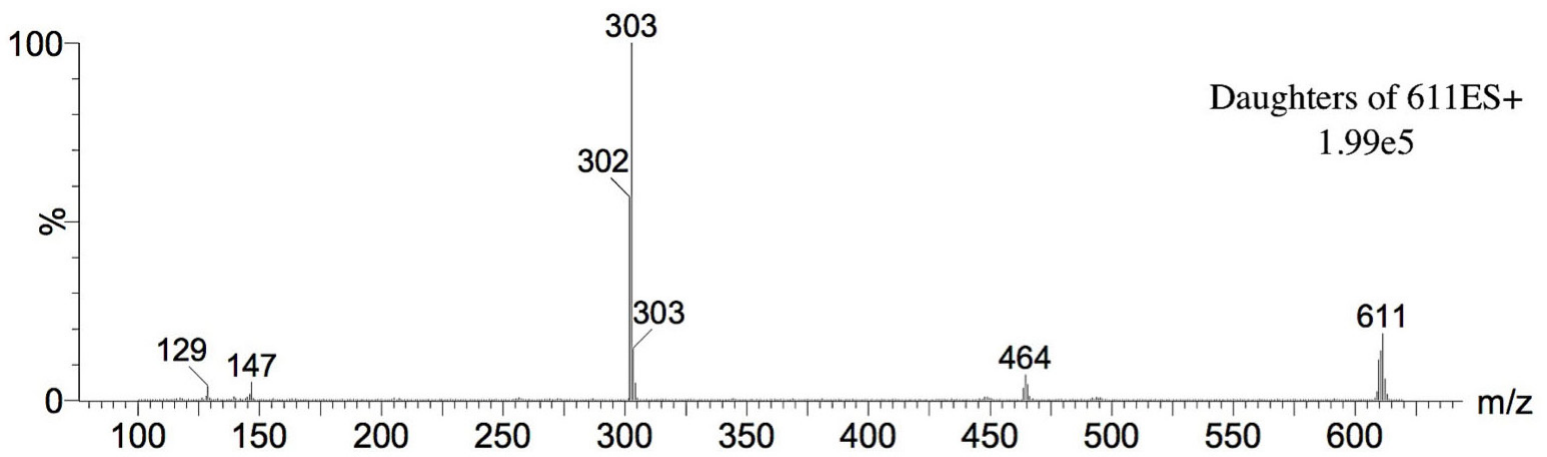
Mass spectrum on ESI positive mode under MRM monitoring for molecular ion m/z 611.

**Fig 11:**
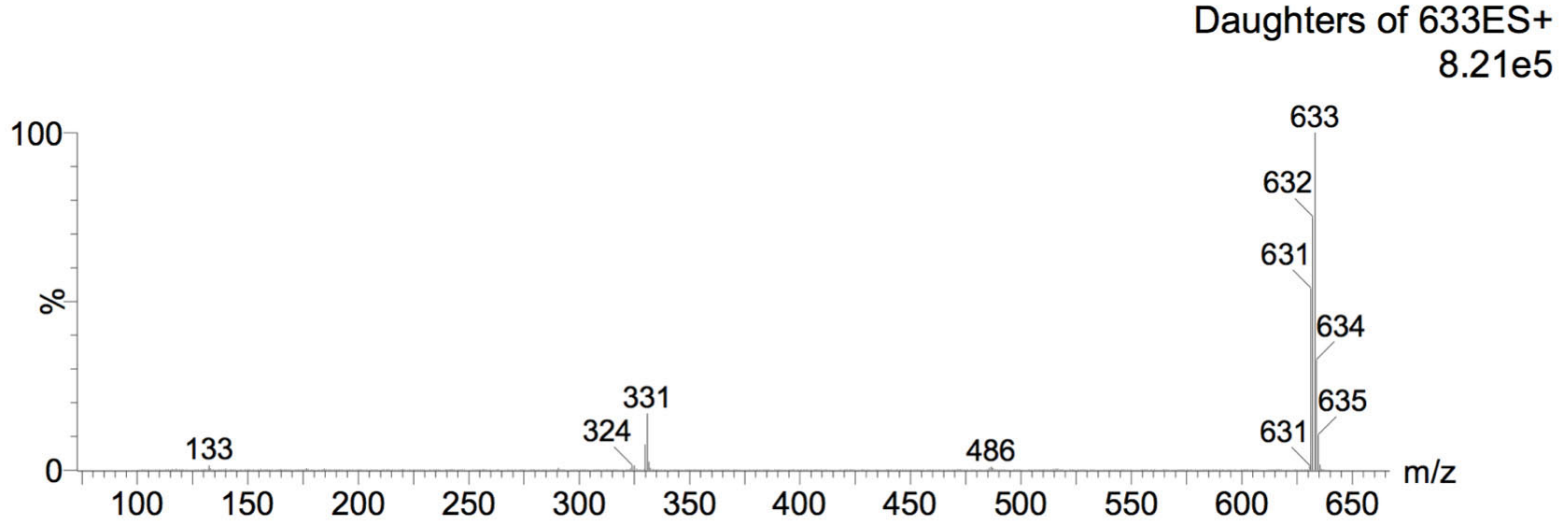
Mass spectrum on ESI positive mode under MRM monitoring for molecular ion m/z 633.

**Fig 12:**
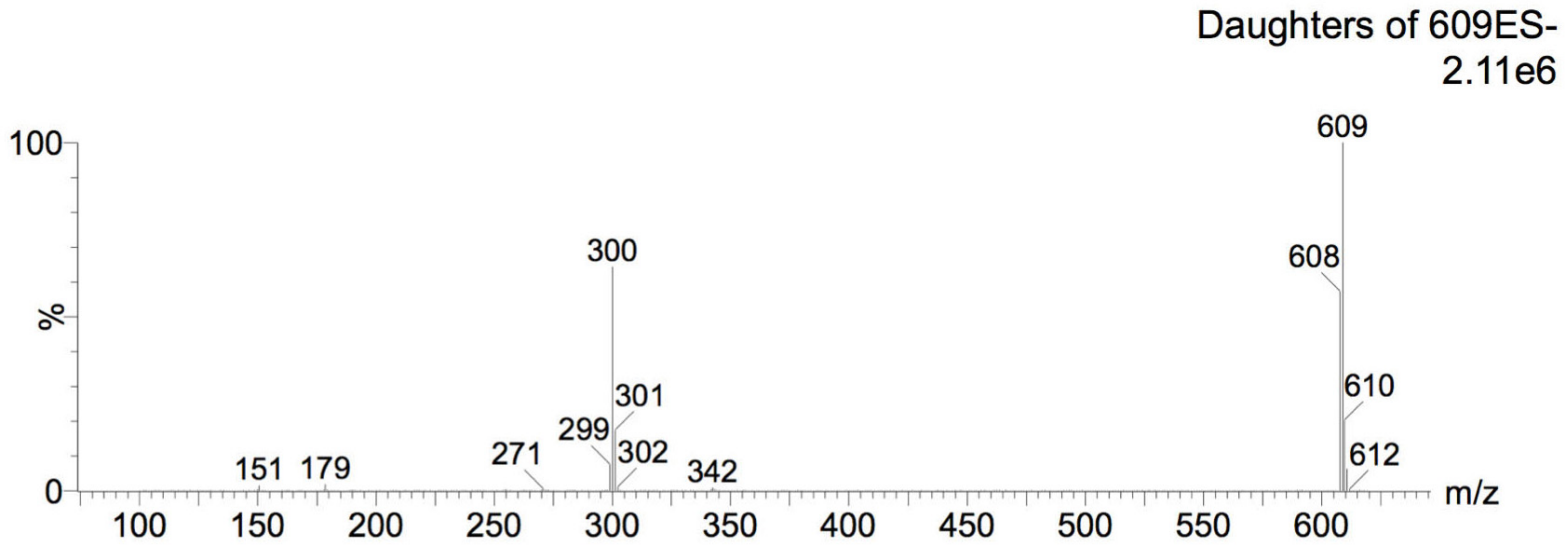
Mass spectrum on ESI negative mode under MRM monitoring for molecular ion m/z 609.

For identity confirmation of rutin, ESI positive and negative modes were employed. For ESI positive mode, protonated ion [M+H]^+^ (m/z 611, Fig 10) and sodiated ion [M+Na]^+^ (m/z 633, Fig 11) as well as their respective product ions were monitored. For ESI negative mode, the deprotonated ion [M-H]^-^ (m/z 609, Fig 12) and its respective product ions were monitored.

## Discussion

The complexity of non-resolving inflammation and the lack of understanding it render pharmacological therapy difficult [1]. Multipronged therapeutic approaches are needed to treat chronic inflammatory diseases, and the most potent drug interventions may increase the risk of adverse effects [28]. Glucocorticoids are commonly used as a reference drug when treating chronic inflammation, and their major adverse effect is additional bone loss [29].

Many epidemiological and experimental studies suggest that natural products may have the ability to decrease oxidative and inflammatory processes, and that their use as dietary products can help prevent chronic diseases [30]. The MSE and fractions were primarily tested for anti-inflammatory effects using the neutrophil migration model as an *in vivo* activity screening model. These findings showed that the extract and all the fractions, except the HF, were to reduce the number of neutrophils migrating into the carrageenan-induced peritonitis. However, with the bioguided method of fractionation, we were able to establish that the AF concentrated the active compound and presented the most significant anti-inflammatory reduction. The mechanisms involved in the inhibition of neutrophil migration to the damage site are accompanied by the reduction in inflammatory markers and oxidative stress [31]. The AF promoted the significant reduction of cytokine IL-1β, which is involved in the release of prostanoids [32]. The release of pro-inflammatory cytokines is related to the migration process, and induces the rolling and adhesion of neutrophils in the vascular endothelium and transmigration to the inflammatory site [1]. The results revealed that IL-1-1β, but not TNF-α, was suppressed, and they have different functions even when they present many proinflammatory-shared activities [33]. Using a chronic inflammatory *in vivo* model, authors support the main role of TNF-α being linked to mediating inflammation and IL-1β controlling bone metabolism, its destruction and inflammation properties [33,34]. In this way, the putative mechanism associated with this activity may be due to the inhibition of the synthesis of cytokines such as IL-1β, which are involved in tissue degradation, as well as to inflammatory process interfering with cell migration [35].

The carrageenan paw edema was accessed to verify the potency of the AF in the inflammation process [36]. The biphasic nature of edema is key in the role of the inflammatory response [37]. In the first phase (0-3h) there is an increase in histamine, serotonin and chemical mediators, and these are related to the greater vascular permeability and production of cytokines such as IL-1β and TNF-α [23]. The second phase of the edema is characterized by the recruitment of prostaglandins, proteases, mediators and most of the available anti-inflammatory drugs are useful to suppress it [38]. The results of the AF treated orally (30 mg/kg) showed an anti-inflammatory activity equal to the positive control indomethacin given intraperitoneally (10 mg/kg). In this way, the anti-inflammatory effect of the fraction may be due to the suppression of the cyclooxygenases involved in the formation of prostaglandins.

This study demonstrated that the AF and EAF had a significant inhibitory effect on MMP-9 accessed by zymography. MMP-9 (gelatinase B, 92 KDa Type IV) is produced in the cell environment and activated after release into the extracellular space [9]. This protein is involved in the breakdown of the extracellular process of bone development and may be found in many pathological conditions, such as arthritis and tumor metastasis [39]. The use of natural products to inhibit MMPs may contribute to attenuating the proteolysis of the extracellular matrix and the role in bone osteoclast resorption [40].

Osteoclasts are formed by the fusion of hematopoietic cells of monocyte-macrophage lineage during the differentiation process [41]. RAW 264.7 cells are precursors and can express phenotype marker genes such as *TRAP* and *Ctsk*, which represent the expression of mature osteoclasts [42]. The AF was able to reduce the expression of both genes and the enzyme CAII, which is overexpressed during the bone resorption process. Despite the lack of scientific information about natural products and bone-protective effects, it is known that flavonoids, a common class of natural products, have shown promise in the area of health promotion related to dietary components like calcium and vitamin D [43]. Flavonoids have been related to the activity of the signaling pathways that influence the osteoblast in an osteoclast difference. Consistent with this study, authors have tested an osteoclast differential *in vitro* model on RAW 264.7 cells and reported a therapeutic potential in a traditional Chinese herb for reducing the *TRAP*, *MMP-9*, *Ctsk* genes in this way, showing an ability to interact directly with the bone cells (osteoblasts, osteoclasts, osteocytes) [44].

Some tests that evaluate radical scavenging capacity may contribute to the etiology of various degenerative diseases, particularly those related to the chronic inflammation process [28]. When sequestering, radical antioxidants can regulate the oxidation pathway [45]. The ABTS and DPPH assays conducted on *M*. *sylvestris* and fractions screened the biologically antioxidant activity and related it to the bone remodeling effect. In a previous report, it was indicated that same *M*. *sylvestris* extract and fractions contained several classes of phenolic compounds that may be responsible to antioxidant effects [21]. Thus, authors demonstrated a linear correlation between the phenolic content and antioxidant effects [46]. In agreement with this study, other investigations have reported the highest antioxidant activity for the AF using the β-carotene-linoleic acid method [16]. In particular, the literature shows that extracts obtained from leaves, as MSE were, have a very strong antioxidant property and the activity is tied to the region where the plant was collected, with the strongest obtained from *M*. *sylvestris* extracts found in northeastern Portugal [18].

The chemical analysis of the AF identified the bioactive compound rutin. This compound is a common flavonoid used in plant-derived beverages, food and folk medicine. Studies have reported diverse pharmacological activities of rutin, including promoting health and reducing risk of chronic diseases. In particular and consistent with this study, research has found rutin to be an anti-inflammatory drug candidate with a unique mechanism for selective COX-2 [47]. Authors have also reported that rutin could inhibit more than 20 genes coding for critical pro-inflammatory factors including TNF-α, IL-1β, IL-1 and IL-8, and migration inhibitory factor [48]. Rutin appears to be a potential phytochemical ingredient for chronic inflammatory treatment or even a promising functional food for the market.

## Conclusion

The AF of *M*. *sylvestris* presented the capacity for anti-inflammatory, anti- oesteoclasteogenic and antioxidant activities. In addition, we suggest that the bioactive fraction and its major compound rutin may be a good candidate for drug discovery in the therapy of chronic inflammatory diseases.
